# Coordination and Synchronisation of Anti-Predation Vigilance in Two Crane Species

**DOI:** 10.1371/journal.pone.0026447

**Published:** 2011-10-19

**Authors:** Chen Ge, Guy Beauchamp, Zhongqiu Li

**Affiliations:** 1 The State Key Laboratory of Pharmaceutical Biotechnology, School of Life Science, Nanjing University, Nanjing, Jiangsu, China; 2 Faculty of Veterinary Medicine, University of Montréal, St-Hyacinthe, Québec, Canada; University of Bristol, United Kingdom

## Abstract

Much of the previous research on anti-predation vigilance in groups has assumed independent scanning for threats among group members. Alternative patterns that are based on monitoring the vigilance levels of companions can also be adaptive. Coordination of vigilance, in which foragers avoid scanning at the same time as others, should decrease the odds that no group member is alert. Synchronisation of vigilance implies that individuals are more likely to be vigilant when companions are already vigilant. While synchronisation will increase the odds that no one is vigilant, it may allow a better assessment of potential threats. We investigated temporal sequences of vigilance in family flocks consisting of two parents and at most two juveniles in two species of cranes in coastal China. We established whether the observed probability that at least one parent is alert was greater (coordination) or lower (synchronisation) than that predicted under the null hypothesis of independent vigilance. We documented coordination of vigilance in common cranes (*Grus grus*) foraging in an area with high potential for disturbance by people. We documented synchronisation of vigilance in red-crowned cranes (*Grus japonensis*) in the less but not in the more disturbed area. Coordination in small flocks leads to high collective vigilance but low foraging rates that may not be suitable in areas with low disturbance. We also argue that synchronisation should break down in areas with high disturbance because periods with low vigilance are riskier. Results highlight the view that temporal patterns of vigilance can take many forms depending on ecological factors.

## Introduction

Vigilance is used by animals to monitor potential threats related to predators or conspecifics [1]. It has long been known that individuals can reduce their vigilance levels in groups as a result of increased corporate vigilance and diluted predation risk [2]. However, the temporal organization of vigilance bouts in groups has received less attention. Earlier models of vigilance assumed for simplicity that the probability that an individual interrupts feeding to become vigilant is constant through time and independent of the level of vigilance maintained by companions in the group [3]–[5]. With these assumptions, sequences of vigilant and non-vigilant bouts become unpredictable through time and unique to each individual [6]. The lack of synchrony in vigilance among individuals that results from independent vigilance is important because corporate vigilance would not be higher in groups if all individuals were vigilant at the same time.

Independent vigilance can be contrasted with two other vigilance strategies. Coordination represents a strategy where individuals alternate vigilance and thereby minimize the amount of time where no one is actually vigilant [7]. Due to chance, occurrences when no one is vigilant may be quite common when individuals scan independently, especially in small groups, making groups temporarily very vulnerable to attack. Coordination may be achieved by paying attention to the vigilance state of neighbours and being more vigilant when fewer companions are vigilant. If we calculate the probability that at least one individual in the group is vigilant, which is one component of collective vigilance [8], this probability in coordinated groups should be higher than predicted by independent scanning. Coordination has only been documented qualitatively so far in sentinel systems, where individuals take turn being vigilant for the whole group usually from a vantage point [9]–[11]. Alternated vigilance has also been documented in paired birds where males maintain vigilance while females feed [12]. It is thought that the prohibitive cost of monitoring neighbours may explain why coordination is not more common in nature [7]. Nevertheless, models predict that coordination is more likely in small groups, when direct detection of threats is not reliable when non-overtly vigilant, and when information about threats can pass easily among group members [13]–[15].

With synchronisation, individuals tend to maintain the same vigilance state as their neighbours producing periods where all individuals are vigilant at the same time. Synchronisation also requires that individuals pay attention to what their neighbours are doing but this time individuals tend to copy what the others are doing. With synchronisation, the probability that at least one individual is vigilant in the group is expected to be lower than predicted with independent scanning [16]. Synchronisation, through copying, may be useful in several ways. With synchronisation, animals can avoid being the least vigilant should a predator attack [17]. Synchronisation may allow more individuals to assess a threat at the same time, making it easier to reach a consensus about the best course of action [18]. Synchronisation of vigilant periods may also occur indirectly in response to synchronisation of foraging effort [19]. Evidence for synchronisation is more common than for coordination [16], [20]–[25] and strongest when it is possible to eliminate the simple alternative that all animals are vigilant at the same time because they have all detected the same threat independently [26].

Here, we examine the relevance of these three vigilance strategies in two species of cranes wintering in coastal China. Wintering common cranes (*Grus grus*) and red-crowned cranes (*Grus japonensis*) are ideal to investigate temporal vigilance patterns. Cranes are large birds that face few threats outside human disturbances. With such specific sources of disturbance, cranes are not readily alarmed, reducing the likelihood of synchronisation caused by stimuli that occur outside the group. Therefore, it is possible to obtain long sequences of observations without real or imagined threats.

The basic unit of social organization in these two wintering crane species is a pair of adult birds with between 0–2 juveniles [27], [28]. Cranes in families forage in small compact groups where communication about threats should be facilitated. These factors should favour coordination of vigilance. For the red-crowned crane, we also contrasted vigilance patterns in two areas that differ in the level of human disturbances. We predicted that synchronisation should be least likely in the area with the most potential for disturbances because it would lead more often to periods where no one in the group is vigilant.

## Methods

### Study Area and Animals

We conducted the study in the Yancheng National Natural Reserve for Coastal Rare Birds (32°36′–34°28′ N, 119°51′–121°5′ E), which is located on the coastline of central Jiangsu Province, China. The reserve is divided into three zones of differing conservation priorities, including a core zone of 219 km^2^, with highest conservation priorities, a buffer zone of 557 km^2^, with medium priority, and an experimental zone of 2066 km^2^, with lower priority. In the buffer zone, human intrusions can be frequent as farmers plant crops and people visit the reserve. In contrast, the core area has restrictions on the number of visitors and farming is not allowed, thus reducing the level of disturbance caused by human activities in this zone.

The study was conducted in the core and buffer zones from the winter of 2009 to the spring of 2011. We focused on two winter migratory crane species in the reserve: red-crowned crane and common crane. The red crowned crane, which is listed as a First-Grade State Protection animal in China and as an Endangered Species in IUCN Red List, usually gathers in small family flocks consisting of two adult birds and between 0–2 juvenile birds. The common crane, listed as a Second-Grade State Protection animal in China and as a species of least concern in IUCN Red List, usually form much larger flocks, but the basic unit is still a family of two adults and between 0–2 juveniles. The red-crowned crane shows a preference for grassland, which is commonly found in the core zone [29], while the common crane usually forages in farmland that occurs in the buffer zone.

In these habitats, the two crane species feed on left-over grains or naturally-occurring seeds as well as small insects. When foraging, cranes occur mostly in family units. We have noted very few instances of aggression within crane families but families may interact with one another. Density of birds in red-crowned cranes was quite low and families were often spread out in the habitat. In common cranes, density in agricultural fields can be quite high. To control for density effects, we focused on families that were foraging apart from the others minimising any potential influence of the rest of the flock.

### Data Collection

We focused on discrete family flocks consisting of two adult cranes and between 0–2 juveniles. In the winter, juvenile cranes are almost adult size but can still be identified through plumage characteristics. We could not distinguish between males and females because they have similar body size and plumage. Crane families were located during regular surveys of the reserve. The same route was not used more than once on the same day to avoid sampling the same flocks. Observations were not carried out on days with rain, snow or strong winds to lessen bias caused by extreme weather. We conducted observations from early morning to late afternoon mostly in January and February.

Once a foraging crane family was located, we used focal sampling to record behaviour. Using binoculars or a spotting scope, we determined whether each bird in the flock was vigilant or not every minute for 30 min or until the flock flew away or changed in size. Vigilance refers to a crane stretching the head upwards while standing erect or scanning around. We could keep track of the identity of each bird during sampling given that individuals did not move to a large extent while foraging. Typically, observations were carried 100–300 m away to reduce potential disturbances by observers. We carried out observations during periods where birds were not disturbed by people. In family flocks, an adult bird typically scans once every minute for about 20 s [28], which makes the timing of our sampling procedure reasonable.

### Data Analysis

The percentage of scans with vigilance served as our metric of vigilance and was arcsine square-root transformed prior to statistical analyses. First, we established whether individual vigilance in red-crowned cranes varied as a function of age, zone and family size. In common cranes, zone was not included since most birds foraged in the buffer zone. We used a linear mixed model with flock id as a random factor. We then performed an analysis restricted to the buffer zone to compare vigilance in the two species.

Using the sequence of behavioural observations, we established collective vigilance considering adults only since vigilance levels by the juveniles were quite low (see below). Collective vigilance considering adults only was measured as the percentage of scans in which at least one of the two adults was vigilant. For each species separately, we examined whether collective vigilance varied as a function of zone and family size using linear models.

We compared observed and predicted collective vigilance separately for each species. The predicted level of collective vigilance was calculated assuming that each individual scans for threats independently of the others. The predicted collective vigilance is given by 100-[(100-*P*1)*(100-*P*2)], where *P* represents the percentage of scans where adult 1 or adult 2 is vigilant. We calculated the contrast between observed and predicted values for each flock. A positive value indicates that individuals tend to coordinate their vigilance bouts (too few sequences where individuals scan at the same time), while a negative value indicates synchronisation (too many instances where individuals scan at the same time). To examine synchrony and coordination between adults and juveniles, we also calculated observed and predicted collective vigilance considering one parent and one juvenile randomly selected from each flock with at least one juvenile. In all cases, we used a paired *t*-test to determine whether observed and predicted values differed significantly from one another.

Sexual differences have been noted in other crane species for vigilance levels [30]. Since we could not identify sex for our focal subjects, the random selection procedure ensured that at the very least we did not choose one sex more often than the other. However, we cannot tell whether patterns of collective vigilance are similar for males and females.

## Results

We conducted a total of 92 focal scans. Flock size ranged from three to four in common cranes (n = 26) and from two to four in red-crowned cranes (n = 66). Focal scans lasted on average 28.6 min (range: 13 to 32 min), which is very close to the maximum set time. Red-crowned crane focals took place primarily in the core zone (n = 55). All focals with the common crane took place in the buffer zone, and we discarded the five focals that were recorded away from farmland to increase homogeneity in our sample.

### Individual and collective vigilance

**Figure 1:**
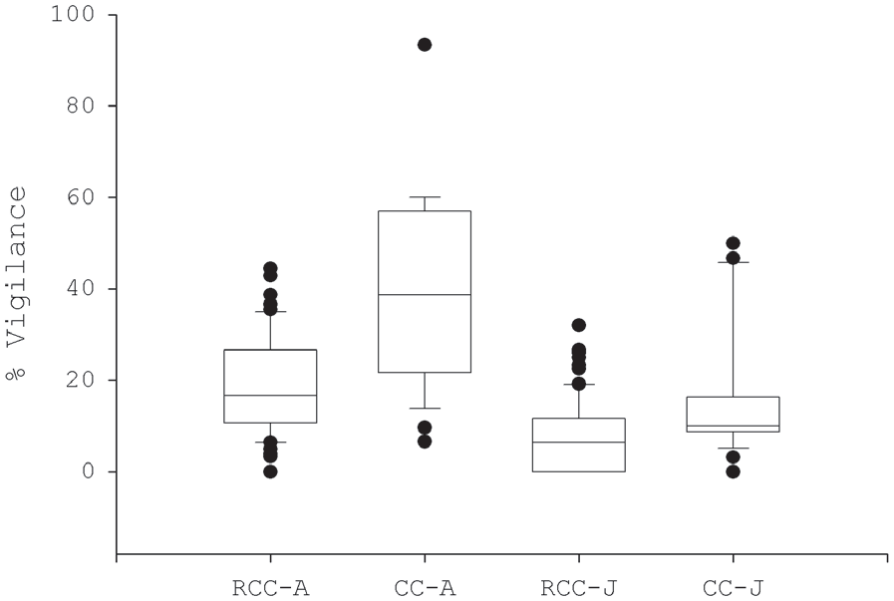
Percentage of scans with vigilance in family flocks of two crane species. Vigilance is shown for adults (A) and juveniles (J) in red-crowned crane (RCC) and common crane (CC).

Individual vigilance in common cranes did not vary as a function of family size (F_1,20_ = 0.32, p = 0.17) but was higher in adults than juveniles (F_1,20_ = 24.0, p<0.0001; fig. 1). In red-crowned cranes, vigilance did not vary as a function of family size (F_2,45_ = 0.10, p = 0.91) and between zones (F_1,45_ = 0.02, p = 0.88) but was higher in adults than juveniles (F_1,45_ = 22.0, p<0.0001; fig. 1). In the buffer zone, where both species co-occurred, vigilance was higher in common cranes than in red-crowned cranes (F_1,56_ = 5.9, p = 0.02), controlling for age and family size.

Considering adults only, collective vigilance was on average 57.7% (range: 16.7–93.8%) in common cranes and 32.8% (range: 10.0–57.1%) in red-crowned cranes. In common cranes, collective vigilance did not vary with family size (F_1,19_ = 1.7, p = 0.22). In red-crowned cranes, collective vigilance did not vary with family size (F_2,62_ = 1.4, p = 0.26) but was higher in the buffer zone than in the core zone (F_1,62_ = 1.6, p = 0.03).

### Comparison of observed and expected collective vigilance

**Figure 2:**
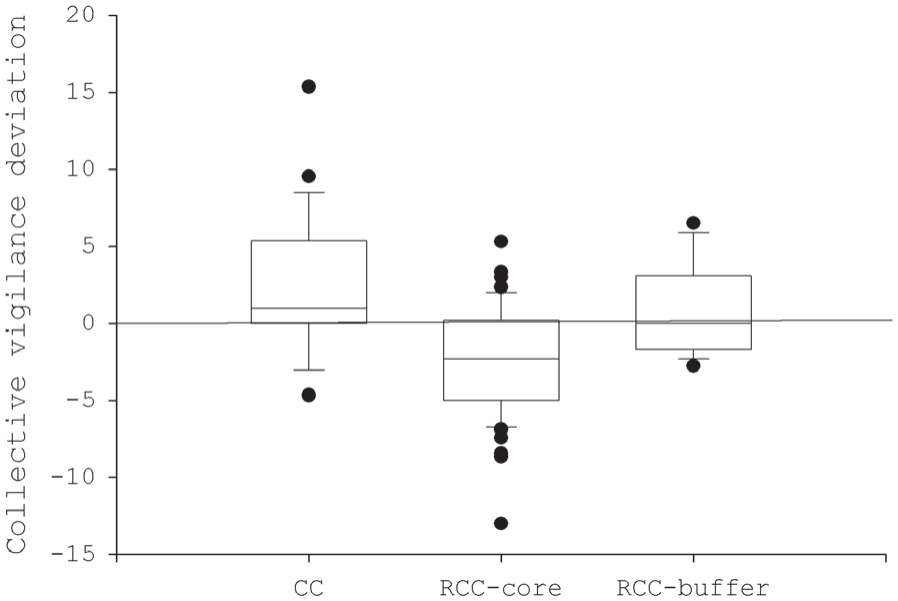
Comparisons of observed and expected collective vigilance between adults in two crane species. Collective vigilance was calculated using the two adults of each flock. A positive contrast value indicates coordinated vigilance while a negative value indicates synchronised vigilance (CC: common crane; RCC-core: red-crowned crane in the core zone; RCC-buffer: red-crowned crane in the buffer zone).

First, we consider collective vigilance calculated with adults only. In common cranes, observed values were significantly larger than expected values (t_20_ = 2.4, p = 0.03; fig. 2). In red-crowned cranes, observed values were significantly smaller than expected values in the core zone (t_54_ = −5.2, p<0.0001) but not in the buffer zone (t_10_ = 0.83, p = 0.43; fig. 2).

**Figure 3:**
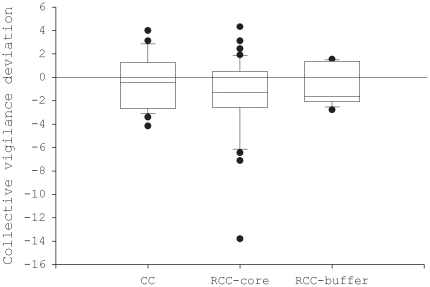
Comparisons of observed and expected collective vigilance between adults and juveniles in two crane species. Collective vigilance was calculated using one adult and one juvenile in each flock. A positive value indicates coordinated vigilance while a negative value indicates synchronised vigilance (CC: common crane; RCC-core: red-crowned crane in the core zone; RCC-buffer: red-crowned crane in the buffer zone).

For collective vigilance calculated with one adult and one juvenile, observed values were not significantly different than expected values in common cranes (t_20_ = −0.74, p = 0.47; fig. 3). In red-crowned cranes, observed values were significantly smaller than expected values in the core zone (t_36_ = −2.8, p = 0.009) but not in the buffer zone (t_8_ = −1.2, p = 0.26; fig. 3).

## Discussion

We found evidence for coordination of vigilance in common cranes and for differential level of synchronisation of vigilance in red-crowned cranes as a function of habitat. We first focus on general features of vigilance in the two crane species.

In the small family flocks of both species, adults were more vigilant than juveniles and did not adjust vigilance levels according to family size. In crane family flocks, adults are more experienced than juveniles and forage with greater efficiency, which may explain why juveniles were less vigilant than adults [27], [30]–[32]. In red-crowned cranes, collective but not individual vigilance was higher in the area with more disturbances. Previous work with this species, but not restricted to families, documented a large increase in individual vigilance in the buffer zone controlling for flock size, supporting the hypothesis that increased disturbances lead to greater vigilance [28].

Common cranes usually gathered in dense flocks consisting of hundreds of individuals. Small population size restricts the size of the foraging red-crowned crane flocks, which are typically found in smaller flocks than common cranes even in the buffer zone [32]. Higher vigilance in common cranes than in red-crowned cranes in the buffer zone partly reflects the greater potential for foraging disruptions caused by the presence of large numbers of potential competitors [31], [33].

We turn now to the temporal organization of vigilance in the two crane species. This is the first study, to our knowledge, to document coordination of vigilance quantitatively in foraging groups without sentinels. Small and compact groups are thought to favour coordination of vigilance [13]–[15]. Coordination is also probably enhanced by the fact that parent cranes are also vigilant to protect their young. Such coordination allows higher levels of collective vigilance that could be achieved through independent scanning or synchronisation. Indeed, collective vigilance in common cranes was about twice as high as that shown by red-crowned cranes with synchronised vigilance. Small family flocks are common in winter in many species of birds including geese and swans, and it may be worthwhile looking for coordination of vigilance in such flocks [34], [35].

It is noticeable that red-crowned cranes did not show coordination of vigilance in similar family flocks. In contrast to common cranes, red-crowned cranes foraged mostly in grassland areas of the core zone. Grassland areas in the core zone are characterized by lower disturbance levels but also by lower food availability, as opposed to farmland of the buffer zone with crop leftovers. Low vigilance levels in grassland areas thus reflect low predation risk and a greater emphasis on foraging. We suggest that coordination of vigilance can be costly in such habitats. This is because coordination of vigilance, in groups of two adults, implies that on average only 50% of available time can be allocated to feeding. Coordination of vigilance in such groups should thus break down to allow more foraging in safer habitats. In larger groups, coordination, in theory, would allow individuals to maintain lower vigilance levels while still benefiting from high collective vigilance. We did not document vigilance patterns in larger groups but we note that coordination is thought less likely in such groups [13]–[15]. Coordination of vigilance could be explored in larger groups of the common crane to assess this hypothesis in future work.

Instead of coordinating vigilance, red-crowned cranes actually synchronised their vigilance. Synchronisation can arise when predators show a preference for stragglers [17], individuals adopting low vigilance levels when other companions are more alert. Such an explanation appears unlikely in large red-crowned cranes that have few natural predators. Synchronisation caused by the presence of external stimuli also appears unlikely in cranes where few external threats, besides human intrusion, are possible.

Synchronisation of vigilance can be caused by copying the behaviour of neighbours [36]–[38]. An advantage of copying is that it allows individuals to use one another as a real-time source of information about disturbances. Indeed, the vigilant posture of a companion may indicate to others that a threat is imminent and worth investigating or conversely that the situation is safe if the others are not vigilant. Copying makes sense for individuals that have little knowledge of potential threats such as the juveniles here in their first winter. We have partial support for this prediction. Juveniles synchronised their vigilance with their parents in red-crowned cranes from the core zone. A recent study documented synchronisation of vigilance among adults in a brood-rearing species but did not investigate synchronisation between adults and juveniles [25].

Copying makes more sense when the level of risk is low rather than high. Vigilant individuals may be a source of information about predation risk but also a source of error if they evaluate the situation mistakenly [39]. Copying errors carries more costs when predation risk is high because a real threat is more likely to be missed. A further cost of copying is that it increases the risk that no one is vigilant at any given time. These two factors may explain why red-crowned cranes avoided synchronisation of vigilance in the buffer zone. Collective vigilance in the buffer zone was also higher due to lack of synchrony and the more random timing of vigilance may be more appropriate to the higher level of risk in this habitat.

Why did red-crowned cranes fail to coordinate their vigilance in the buffer zone? Coordination would have lead to higher collective vigilance as in common cranes in this riskier zone. Interestingly, we documented lower vigilance levels in red-crowned cranes than in common cranes in the buffer zone. This can partly be explained by the fact that red-crowned cranes in the buffer zone rarely fed in farmland but rather in reed beds where reed can serve as shelter [29]. In reed beds, human intrusion level is also relatively lower than in farmland. The lower perception of risk in reed beds, relative to farmland, may have favoured the lower collective vigilance afforded by independent scanning. It would be worthwhile investigating vigilance in the two species of cranes foraging in the same habitat. We also note that coordination of vigilance requires good visual communication between group members [14], [15] in the absence of vocal communication [40]. Communication may be more difficult to maintain in densely vegetated reed beds than in more barren farmland.

Given that vigilance is mostly performed at the expanse of foraging, alternative mechanisms for coordination and synchronisation of vigilance may focus on foraging constraints rather than management of predation risk. For instance, coordination of vigilance may arise to some extent if some individuals in the group monitor companions that are feeding head down for opportunities to exploit their food discoveries [41]. However, the two crane species here exploit very small food items in this agricultural landscape, such as grain and insects, which cannot be stolen. Synchronisation of vigilance may also reflect indirectly synchronisation of foraging effort. Pelicans (*Pelecanus* spp.), for example, fish cooperatively and dip their head in the water at the same time [42] so that vigilance is indirectly performed synchronously as well. However, cooperative foraging is not needed in cranes and individuals can obtain their small food items alone. Nevertheless, we stress that it is important to address in future similar work foraging mechanisms that can cause apparent coordination or synchronisation of vigilance.

Results from this study highlight the view that temporal patterns of vigilance can take many forms depending on ecological factors. As shown here, the precise pattern documented in one species may depend on group size, group density and the ability to extract information from the behaviour of companions.
